# Theory of Mind impairment in childhood narcolepsy type 1: a case–control study

**DOI:** 10.1093/braincomms/fcae063

**Published:** 2024-02-27

**Authors:** Marco Veneruso, Paola Del Sette, Ramona Cordani, Serena Lecce, Fabio Pizza, Lorenzo Chiarella, Cristina Venturino, Lino Nobili, Giuseppe Plazzi

**Affiliations:** Dipartimento di Neuroscienze, Riabilitazione, Oftalmologia, Genetica e Scienze Materno-Infantili, Università di Genova, 16132 Genova, Italy; UOC Neuropsichiatria Infantile, IRCCS Istituto Giannina Gaslini, 16147 Genova, Italy; UO Psicologia Clinica e Psicoterapia, IRCCS Ospedale Policlinico San Martino, 16132 Genova, Italy; Dipartimento di Neuroscienze, Riabilitazione, Oftalmologia, Genetica e Scienze Materno-Infantili, Università di Genova, 16132 Genova, Italy; UOC Neuropsichiatria Infantile, IRCCS Istituto Giannina Gaslini, 16147 Genova, Italy; Dipartimento di Scienze del Sistema Nervoso e del Comportamento, Università di Pavia, 27100 Pavia, Italy; Dipartimento di Scienze Biomediche e Neuromotorie, Alma Mater Studiorum—Università di Bologna, 40126 Bologna, Italy; UOC Clinica Neurologica, IRCCS Istituto delle Scienze Neurologiche di Bologna, 40139 Bologna, Italy; Dipartimento di Neuroscienze, Riabilitazione, Oftalmologia, Genetica e Scienze Materno-Infantili, Università di Genova, 16132 Genova, Italy; UOC Neuropsichiatria Infantile, IRCCS Istituto Giannina Gaslini, 16147 Genova, Italy; UOSD Psicologia, IRCCS Istituto Giannina Gaslini, 16147 Genova, Italy; Dipartimento di Neuroscienze, Riabilitazione, Oftalmologia, Genetica e Scienze Materno-Infantili, Università di Genova, 16132 Genova, Italy; UOC Neuropsichiatria Infantile, IRCCS Istituto Giannina Gaslini, 16147 Genova, Italy; UOC Clinica Neurologica, IRCCS Istituto delle Scienze Neurologiche di Bologna, 40139 Bologna, Italy; Dipartimento di Scienze Biomediche, Metaboliche e Neuroscienze, Università di Modena e Reggio Emilia, 41125 Modena, Italy

**Keywords:** narcolepsy, Theory of Mind, cataplexy, emotional processing, neuropsychiatric symptoms

## Abstract

Narcolepsy type 1 is a central disorder of hypersomnolence characterized by excessive daytime sleepiness, cataplexy and other rapid eye movement sleep-related manifestations. Neurophysiological studies suggest that narcolepsy type 1 patients may experience impairment in emotional processing due to structural and functional changes in limbic structures and associated areas. However, the only study exploring narcolepsy behavioural responses found no impairment in the ability to recognize emotions, possibly due to compensatory mechanisms. The present study was designed to fill this gap in the literature by investigating the behavioural impairment related to emotional processing focusing on an advanced socio-cognitive skill, namely Theory of Mind, in paediatric narcolepsy type 1 patients. Twenty-two narcolepsy type 1 children and adolescents (six female; age range: 8.0–13.5) and 22 healthy controls matched for age and sex (six female; age range: 8.9–13.0) underwent a neuropsychological evaluation to assess socio-economic status, verbal abilities, working memory, social anxiety and Theory of Mind via a verbal task (i.e. Strange Stories task) and a visual task (i.e. Silent Films). Narcolepsy type 1 patients were also evaluated for disease severity. Patients exhibited impairment in Theory of Mind skills, as assessed both through both verbal (controls median = 8; patients median = 5; *P* = 0.009) and visual tasks (controls median = 8; patients median = 6; *P* = 0.003), compared to healthy controls. Correlation analyses showed that verbal and visual Theory of Mind was negatively related to narcolepsy severity (*ρ* = −0.45, *P* = 0.035 and *ρ* = −0.52, *P* = 0.012), and daytime sleepiness (*ρ* = −0.48, *P* = 0.025 and *ρ* = −0.45, *P* = 0.038). Our study shows a selective impairment in the Theory of Mind domain in children and adolescents with narcolepsy type 1. In addition, our results highlight a link between symptom severity and Theory of Mind, suggesting that lower Theory of Mind levels are associated with higher symptom severity. Further, longitudinal studies are needed to disentangle the direction of this relation and to disambiguate if narcolepsy severity impaired children’s Theory of Mind or if Theory of Mind skills modulate the severity of narcolepsy symptoms by providing a greater ability to avoid cataplexy.

## Teaser

Veneruso *et al*. report that children and adolescents with narcolepsy type 1 have lower Theory of Mind abilities assessed by verbal and visual tasks compared to healthy controls. This impairment seems to be related to symptom severity. These findings pave the way for new therapeutic strategies in paediatric narcolepsy.

## Graphical Abstract

**Graphical Abstract:**
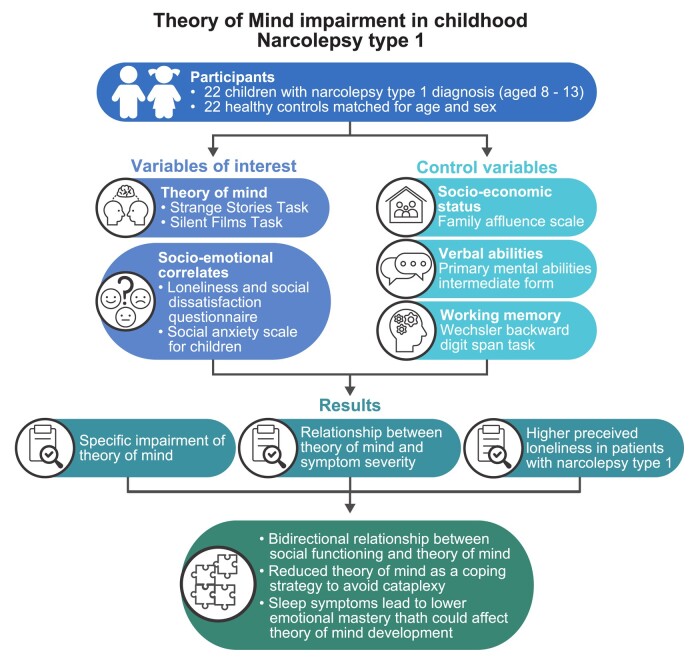

## Introduction

Narcolepsy type 1 (NT1) is a central disorder of hypersomnolence characterized by excessive daytime sleepiness (EDS), cataplexy (i.e. sudden episodes of loss of muscle tone triggered by emotions during wakefulness) and additional rapid eye movement (REM) sleep-related manifestations (sleep paralysis, hypnagogic/hypnopompic hallucinations, disrupted nocturnal sleep and REM sleep behaviour disorder).^1^ NT1 is related to a selective deficiency of cerebrospinal hypocretin-1 (CSF hcrt-1), probably due to an autoimmune process leading to the loss of hcrt-1-producing neurons in the lateral hypothalamus, and is therefore regarded as the *in vivo* model of hypocretin deficiency.^2^

The onset of NT1 typically occurs during childhood or adolescence.^3^ Cataplexy, the pathognomonic symptom of the disease, can be triggered by positive or negative emotional stimuli.^4^

However, the peculiar clinical features of paediatric patients possibly manifesting EDS with behavioural changes and cataplexy as a hypotonic condition also in the absence of clear emotional triggers,^1^ coupled with the possible early onset of concurrent neuropsychiatric symptoms,^5^ can make clinical suspect challenging, often leading to a delay in proper referral and adequate diagnostic workup.^6^

The role of emotions in triggering cataplexy suggests an intrinsic role of emotional processing in NT1 pathophysiology. Among the structures involved in emotion and emotional processing, the limbic system, with particular emphasis on the amygdala, and ventromedial prefrontal cortex, play a central role.^7^

On the other hand, CSF hcrt-1 levels are related to social interactions and subject-reported positive emotions or anger.^8^

Concerning patients with NT1, various studies suggest structural and functional changes in limbic structures, including the amygdala, and associated areas,^9-11^ supporting the existence of a link between emotion network dysfunction and NT1 in both adults and children.^12^

However, there is limited evidence regarding behavioural impairment related to emotional processing. Bayard *et al*.^13^ found no differences in facial emotion recognition between adult subjects with NT1, hypersomnia without cataplexy, and healthy controls, suggesting a possible alternative network, other than the amygdala, to deal with emotional processing. Additionally, de Zambotti *et al*.^14^ found that adult patients’ haemodynamic responses to pictures with positive, neutral, and negative valence mirrored those of healthy controls, but patients reported lower arousal scores and lower valence scores associated with positive stimuli.

No previous studies have evaluated emotional processing and related networks in paediatric subjects with NT1, despite that the high frequency of neuropsychiatric symptoms suggests an impact of childhood-onset NT1 on emotional well-being.^15,16^ Moreover, paediatric patients might show emotional impairment in suppressing their emotion-expressive behaviour in response to emotional stimuli to avoid cataplexy. For these reasons, it might be interesting to investigate behavioural responses to emotional stimuli in younger subjects close to the onset of the disease, in order to reduce the long-term effect of NT1 on emotional development. Moreover, exploring more complex socio-cognitive functions that emotion recognition, namely Theory of Mind (ToM), could provide a more nuanced understanding of the emotional challenges faced by children with NT1. Indeed, ToM is the ability to attribute mental states (including emotions, beliefs, intentions, desires and knowledge) to self or others in order to predict behaviour (Box 1).^17^

Box 1 **Theory of Mind definition and development**

The Theory of Mind (ToM) is a socio-cognitive ability that enables individuals to attribute mental states such as beliefs, desires, emotions and intentions to oneself and others, and to predict social behaviour based on these mental states.^18^

ToM is a complex ability made up of different components that develop at different age-stages from childhood and throughout the lifespan.^19^ The earliest precursor of ToM is children’s pointing. In the first year of life, children start to consider others by imitating their facial expressions and by pointing objects to request something (imperative pointing) and, later on, to draw others’ attention to an interesting object (declarative pointing). After the first year of life, children become more familiar with others’ mind, and they begin to use a mental lexicon, for referring to self and others’ mental states, to appreciate that others have desires and beliefs that can differ from their own, and then to understand that something can be true but someone might not know that.^19^ Between the ages of 3 and 5 years, children face a core developmental shift in their ability to use mental states (beliefs, intentions, knowledge, desires) to explain others’ behaviours. Indeed, they start to understand that others’ behaviours are driven by beliefs that can also be false. Accordingly, at around 4 and 6 years of age, typically developing children usually pass the first and second order false-belief tasks, respectively.^20^ ToM development extend into middle childhood when children become progressively better in understanding others’ false beliefs and mental states within complex social scenarios.^21,22^

ToM and social relationships are intertwined with each other. Evidence in the two directions can be summarized as follows:

- ToM develops during childhood, within the context of social relationships.^23-25^ In support of this claim, individuals with impairments in ToM, such as those with autism spectrum disorder, have difficulty understanding others’ mental states and may struggle with social interactions.^26^ In addition, many studies demonstrated the influence of children’s early social experiences, such as family structures, maternal mind-mindedness and interactions with friends and peers, on later individual differences in ToM.^27^
- Individual differences in ToM play a crucial role in children’s social.^28^ In line with this, longitudinal studies found that difficulties in understanding mental states predict subsequently elevated peer rejection, while mastery ToM predicts later social competences and peer success.^27,29^ Thus, boosting ToM seems to be a simple yet effective way to improve children’s social adjustment.^27^

We hypothesize that children with NT1 may exhibit reduced ToM abilities compared with healthy subjects and that patients’ ToM performances may be related to symptom severity.

## Materials and methods

### Participants

Consecutive children with NT1 diagnosis according to the International Classification of Sleep Disorders Third Edition^30^ aged 8–13 years attending the out-patients clinic at the child neuropsychiatry unit of the Gaslini Institute in Genoa and at the Narcolepsy Center of the Institute of Neurological Sciences in Bologna were recruited.

A group of healthy controls matched for age and sex was randomly selected from a more extensive database of children recruited from primary schools located in Northern Italy. All children had good Italian language proficiency and had no history of developmental delay.

All subjects and their parents gave their written informed consent to participate in the study, which was approved by the Liguria Regional Ethics Committee (453/2022—DB id 12567).

### Experimental procedures

NT1 patients and healthy subjects underwent a battery of tests and questionnaires to assess ToM abilities, as well as possible confounding variables such as socio-economic status, verbal abilities and working memory. Children’s self-rated social anxiety and loneliness were also evaluated as variables of interest.

Children with NT1 underwent a clinical evaluation, including rating scales and questionnaires for the severity of narcolepsy symptoms.

NT1 patients were evaluated during the afternoon in an artificially lighted room after a 15–20-minute nap to minimize the potential effect of sleepiness on performance.

### Theory of Mind measures

ToM was assessed through Strange Stories (SS) task^31^ and Silent Films (SFs) task.^32^

**Figure 1:**
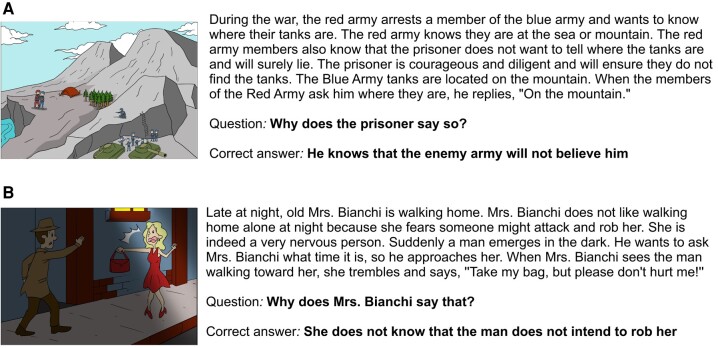
**Examples of items from Strange Stories.** (**A**) Double bluff story. (**B**) Misunderstanding story.

SS task measures children’s ability to understand other’s mental states within complex social scenarios presented in a written form. We administered five mental stories (two double bluffs, two misunderstandings and one persuasion) and three physical/control stories. After each story, children have to explain the reasons behind the main character’s behaviour in a written form. While mental stories require children’s ability to make context-appropriate inferences about the character’s mental states, physical stories require children’s ability to make context-appropriate inferences about the physical (not psychological) cause of the character’s behaviour. According to the scoring guidelines,^33^ 0 points were assigned for incorrect and ‘don’t know’ answers, 1 point for partially correct and implicit answers and 2 points for full and explicit answers. The total mental SS score was calculated by summing the performance obtained at each mental story of the SS task and could range from 0–10. The total physical SS score was calculated by summing the performance at each physical story of the SS task and could range from 0–6 (Fig. 1) (Supplementary Fig. 1).

**Figure 2:**
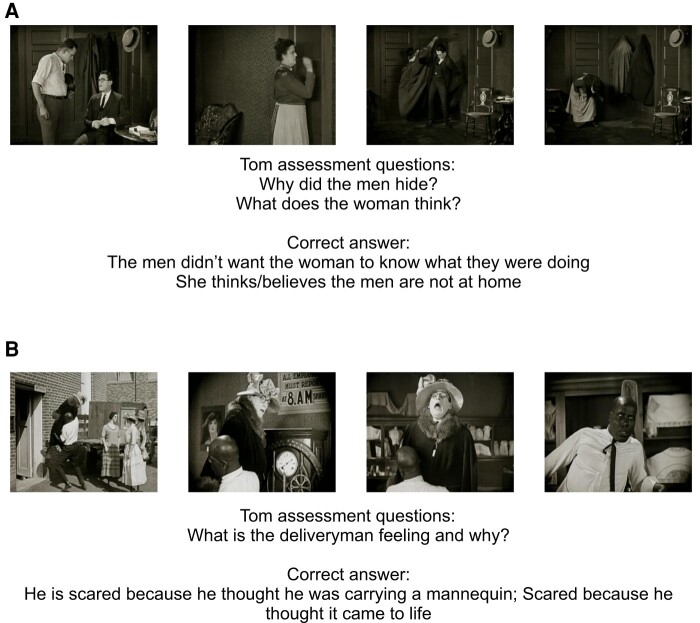
**Screenshots from the Silent Film task.** (**A**) In this scene, we see two men talking in a room. When a woman knocks on the door, the men hide inside the coats hanging on the wall. Once inside the room, the woman looks around and does not see the two men, so she leaves. (**B**) In this scene, we see a delivery man carrying a mannequin that we discover is a man. When the fake mannequin sneezes, the terrified delivery man runs away. Images are from the Harold Lloyd silent film, Safety Last (copyright expired).

SF measures children’s ability to understand others’ mental states based on their behaviour. We administered five short clips (mean length = 25.4 s) from Harold Lloyd’s classic silent comedy, Safety Last. After each clip, children are required to explain the behaviour of the main character. According to the scoring guidelines, 0 points were assigned for incorrect and ‘don’t know’ answers, 1 point for partially correct and 2 points for full and explicit answers. The total SF score was calculated by summing the performance at each clip of the SF task and could range from 0–10 (Fig. 2).

### Socio-emotional correlates

Children’s subjective feeling of loneliness was measured via the Loneliness & Social Dissatisfaction Questionnaire.^34^ The scale comprises 16 target items and five filler items rated on a scale ranging from 1 (‘never’) to 3 (‘always’). Nine items measure the level of perceived social inclusion, four items measure the perceived difficulties of making friends and three items measure the perceived loneliness. Following the scoring guidelines, we calculated three scores based on the sum of the responses to each item: a social inclusion score ranging from 9–27, a difficulty of making friends score ranging from 4–12 and a perceived loneliness score ranging from 3–9.

Social anxiety was measured via the Social Anxiety Scale for Children^35^ (SASC). The scale comprises 10 items rated on a scale ranging from 0 (‘never’) to 2 (‘always’). Six items measure the level of fear about receiving negative evaluation from others and the other four items measure the experience of distress in social situations and deliberate avoidance of those situations. According to the scoring guidelines, we calculated two scores given by the sum at each item: a fear of negative evaluation score ranging from 0–12 and a social avoidance and distress score ranging from 0–8.

### Control variables

We assessed socio-economic status through the Family Affluence Scale,^36^ a short child report on familial (material) wealth. The scale was made by four questions about (i) family car ownership (range: 0–2); (ii) having/not having unshared room (range: 0–1); (iii) number of computers at home (range: 0–3); and (iv) number of times the participants went on a holiday during the past year (range: 0–3). The total score was given by the sum of each response. In the present study, we excluded from the sum the last question (i.e. ‘number of times the participants went on a holiday during the past year’) considering that clinical data collection takes place in a period still affected by travel restrictions due to COVID-19 pandemic (from April 2021–November 2021). Thus, the total socio-economic status score could range from 0–6.

We evaluated children’s verbal abilities via the Italian version of the Vocabulary subtest of the Primary Mental Abilities, Intermediate Form (PMA).^37^ During the task, children had to find in 6 minutes at best the synonyms of 50 target words, choosing among five alternatives. The total verbal abilities score was given by the number of correct responses (possible range 0–50).

We assessed working memory through the WISC-R Backward Digit Span task.^38^ During the task, children were asked to recall seven series of digits, just listened, in reverse order. Series were presented increasing the number of digits (from two to eight) and thus the level of difficulty. A total working memory score was calculated by summing the number of correct answers (possible range 0–7).

### Clinical evaluation

Children with NT1 underwent a clinical evaluation, including an interview designed to investigate the severity of symptoms such as daytime sleepiness, cataplexy, sleep paralysis and hypnagogic hallucinations. In addition, the age of onset of narcolepsy, the diagnostic delay and the use of drugs and behavioural therapy were also assessed.

Epworth Sleepiness Scale for Children and Adolescents (ESS-CHAD)^39^ was administered for the measurement of subjective EDS. The questionnaire consists of eight items rated on a scale ranging from 0 (‘never’) to 3 (‘always’). Each item represents a situation in which patients must rate their chances of falling asleep.

An Italian-translated version of the Pediatric Narcolepsy Severity Scale (PNSS)^40^ was used to measure the subjective severity of narcoleptic symptoms.

The frequency of cataplectic attacks was assessed on a scale from 1–5, where 1 represents one or fewer cataplectic attacks per year, 2 represents more than one cataplectic attacks per year but fewer than one per month, 3 represents one or more attacks per month but fewer than one per week, 4 represents one or more attacks per week but fewer than one per day and 5 represents at least one cataplectic attack per day.^41^

### Statistics

Data were examined for normal distribution performing the Shapiro–Wilk test. Since some variables were not normally distributed and due to the small sample size, we decided to use non-parametric tests.

Group differences were analysed by performing Mann–Whitney U test.

The relationships between ToM and clinical variables were explored with Spearman’s rank correlation coefficient.

Statistical analyses were performed with SPSS version 28.0 for Windows (SPSS, Chicago, USA).

The level of significance was *α* < 0.05.

## Results

### Demographic and clinical characteristics

Demographic and clinical data are presented in Table 1.

**Table 1:** Demographical and clinical characteristics

	Median	Q1–Q3	Range
Age at onset	7.95	6.86–9.28	4.81–10.72
Diagnosis latency	0.65	0.28–1.38	0.04–3.78
Duration of illness	2.81	1.28–4.16	0.98–6.90
ESS-CHAD	13.00	10.75–15.25	7.00–22.00
PNSS	24.00	21.75–30.50	8.00–46.00
Cataplexy frequency	4.00	4.00–5.00	0.00–5.00

ESS-CHAD, Epworth Sleepiness Scale for Children and Adolescents; PNSS, Pediatric Narcolepsy Severity Scale; MASC, Multidimensional Anxiety Scale for Children; CDI, Children’s Depression Inventory; RMET, Reading the Mind in the Eyes Test.

Twenty-two children with NT1 (six female; age, 25% = 9.10, median = 11.4, 75% = 12.1, range: 8.0–13.5) were included in this study. Twenty-two typically developing children were randomly selected from a more extensive database of children recruited from primary schools located in Northern Italy (six female; age, 25% = 9.6, median = 11.5, 75% = 12.6, range: 8.9–13.0). The control sample was matched for age (U = 242.00, *P* > 0.999) and sex.

Patients with NT1 had a median age at disease onset of 7.95 years (SD² 2.47) with a median diagnostic delay of 0.65 years (SD² 0.85). The median disease duration was 2.81 years (SD² 3.15). All patients had CSF hcrt-1 level < 110 pg/mL, and 21 (99.46%) had HLA-DQB1*0602. Five patients (23%) were treatment-naive, with 17 (77%) patients taking drug therapy and 9 (40.9%) using behavioural therapy. Concerning symptom severity, the median score at Pediatric Narcolepsy Severity Scale was 24 (SD² 70.26), the ESS-CHAD median score was 13 (SD² 18.26) and the median score at cataplexy frequency scale was 4 (SD² 1.71), with 2 patients reporting one or fewer cataplectic attacks per year, 2 referring one or more attacks per month but fewer than one per week, 9 reporting one or more attacks per week but fewer than one per day and 9 reporting more than one cataplectic attack per day.

### Theory of Mind

**Figure 3:**
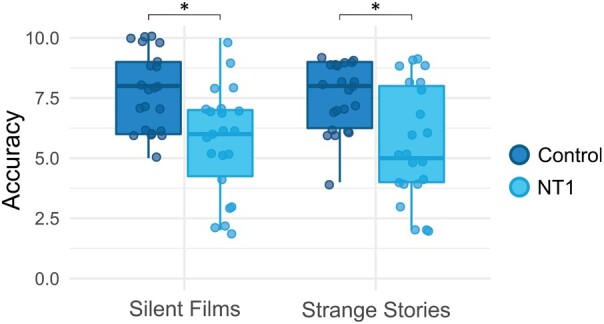
**Theory of Mind tasks differences across the two groups.** Group differences were analysed by performing Mann–Whitney U tests. Patients with narcolepsy type 1 obtained lower scores performing the Strange Stories task for the measurement of the verbal Theory of Mind and the Silent Films task for the measurement of the visual Theory of Mind. Significant differences at *P* < 0.05.

Healthy control children obtained higher scores in ToM measured via mental SS (U = 132.50, *P* = 0.009), and SF (U = 119.00, *P* = 0.003) (Fig. 3). Moreover, there was no difference between groups in physical SS (U = 210.00, *P* = 0.436) (Table 2).

**Table 2:** Group differences within Theory of Mind, socio-emotional correlates and control variables

Healthy controls				NT1				
	Median	Q1–Q3	Range	Median	Q1–Q3	Range	Mann–Whitney U	*P*-value
SES	5.00	3–5	1–6	4.00	3–4.25	1–6	172.50	0.091
Voc	22.00	17.75–28.25	8–33	16.00	12–21	5–31	117.50	0.003**
WM	3.00	2–3	1–4	3.00	3–3	1–5	204.00	0.326
pSS	4.00	4–5	2–6	4.00	3–5	0–6	210.00	0.436
mSS	8.00	6–9	4–9	5.00	4–8	2–9	132.50	0.009**
SF	8.00	6–9.25	5–10	6.00	3.75–7	2–10	119.00	0.003**
LSDQ_SI	11.00	9–13	9–24	11.00	9–12.25	9–19	241.50	0.990
LSDQ_DMF	6.00	4–7	4–10	6.00	4.75–7	4–12	203.50	0.352
LSDQ_PL	3.00	3–3	3–8	4.00	3–5	3–8	151.00	0.016*
SASC_FNE	2.50	1–5	0–9	4.00	1.75–5	0–7	214.00	0.507
SASC_SAD	3.00	2–3.25	0–6	3.00	2–4	0–6	190.5	0.216

SES, socio-economic status; Voc, vocabulary; WM, working memory; mSS, Mental Strange Stories; SF, Silent Film; LSDQ, Loneliness & Social Dissatisfaction Questionnaire; SI, social inclusion; DMF, difficulty of making friends; PL, perceived loneliness; SASC, Social Anxiety Scale for Children; FNE, fear for negative evaluation; SAD, social avoidance and distress.

**P < 0.01. *P < 0.05.

**Figure 4:**
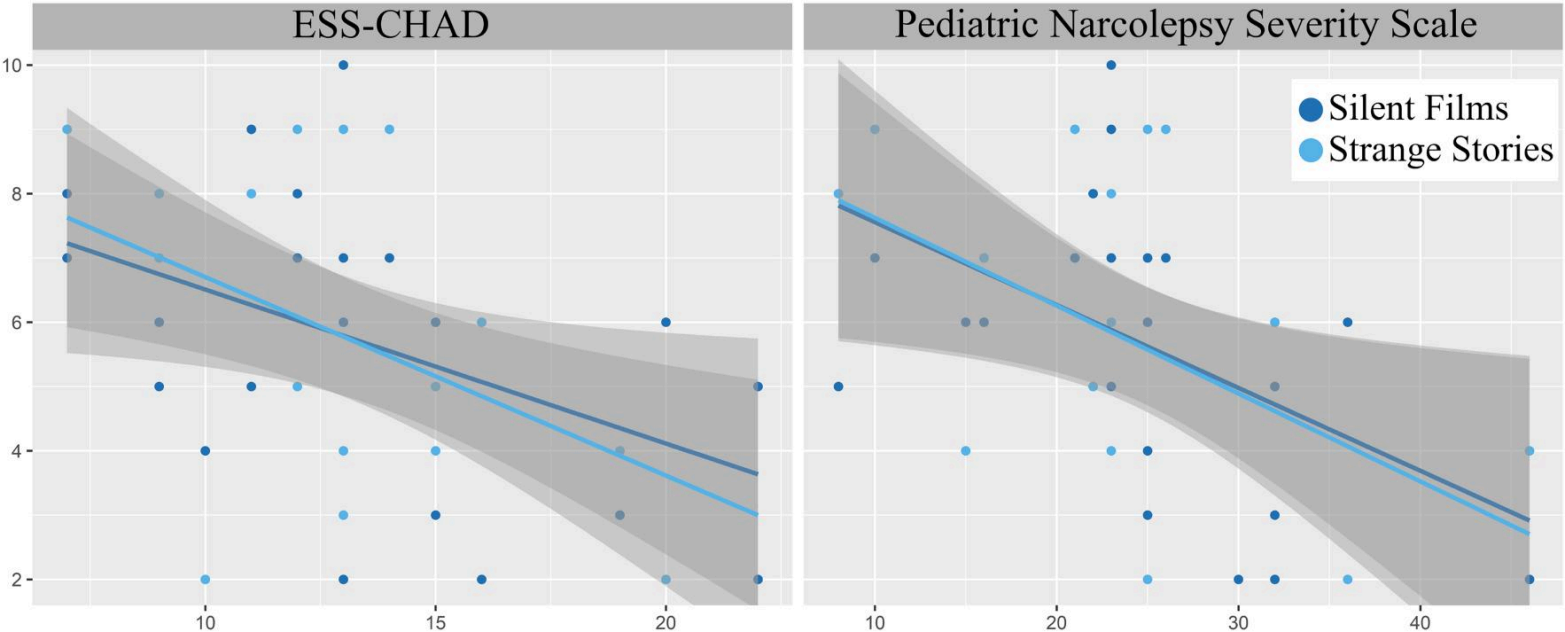
**Correlation between Theory of Mind tasks and clinical variables.** Spearman’s rank correlation coefficient to assess the correlation between both the Strange Stories and the Silent Films task and symptoms severity measured with Pediatric Narcolepsy Severity Scale and Epworth Sleepiness Scale for Children and Adolescents. Significant correlation at *ρ* < −0.4 or *ρ* > 0.4.

Analysing the relationship between ToM and clinical variables, our data showed that ToM, measured with mental SS and SF, negatively correlated with PNSS (*ρ* = −0.45, *P* = 0.035 and *ρ* = −0.52, *P* = 0.012, respectively) and with ESS-CHAD (*ρ* = −0.48, *P* = 0.025 and *ρ* = −0.45, *P* = 0.038, respectively) (Fig. 4).

Conversely, we found no significant correlations between Mental Strange Stories and age at onset (*ρ* = 0.11; *P* = 0.631), diagnostic delay(*ρ* = 0.03; *P* = 0.908), disease duration (*ρ* = 0.26; *P* = 0.249) and cataplexy frequency (*ρ* = −0.25; *P* = 0.255), as well as between SF and age at onset (*ρ* = 0.14; *P* = 0.524), diagnostic delay (*ρ* = 0.15; *P* = 0.496), disease duration (*ρ* = 0.23; *P* = 0.314) and cataplexy frequency (*ρ* = −0.09; *P* = 0.702) (Table 3).

**Table 3:** Correlations between Theory of Mind and clinical variables

	Age at onset	Diagnosis latency	Disease duration	ESS-CHAD	PNSS	Cataplexy frequency
mSS	0.11	0.03	0.26	−0.48*	−0.45*	−0.25
SF	0.14	0.15	0.23	−0.45*	−0.52*	−0.09
SASC-FNE	−0.27	0.03	0.24	−0.11	−0.28	0.19
SASC-SAD	−0.11	−0.26	−0.03	0.10	−0.09	0.16
LSDQ-SI	−0.12	−0.55**	0.30	0.46*	0.45*	−0.05
LSDQ-DMF	−0.30	−0.39	0.27	0.22	0.13	−0.07
LSDQ-PL	−0.05	−0.07	−0.03	−0.25	−0.03	−0.17

mSS, Mental Strange Stories; SF, Silent Film; ESS-CHAD, Epworth Sleepiness Scale for Children and Adolescents; PNSS, Pediatric Narcolepsy Severity Scale; SASC, Social Anxiety Scale for Children; FNE, fear for negative evaluation; SAD, social avoidance and distress; LSDQ, Loneliness & Social Dissatisfaction Questionnaire; SI, social inclusion; DMF, difficulty of making friends; PL, perceived loneliness.

**P < 0.01. *P < 0.05.

### Socio-emotional correlates

We found no difference between groups in perceived social inclusion (U = 241.50, *P* = 0.990), perceived difficulties of making friends (U = 203.50, *P* = 0.352), fear of negative evaluation (U = 214.00, *P* = 0.507) and social avoidance (U = 190.50, *P* = 0.216). Children with NT1 showed higher perceived loneliness than healthy controls (U = 151.00, *P* = 0.016) (Table 2).

We also found that social inclusion correlated negatively with diagnosis latency (*ρ* = −0.55, *P* = 0.008) and positively with PNSS (*ρ* = 0.45, *P* = 0.037) and with ESS-CHAD (*ρ* = 0.46, *P* = 0.031) (see Table 3).

### Control variables

Concerning control variables, we found that patients have fewer vocabulary skills compared to control (U = 117.50, *P* = 0.003), while there were no differences between groups in socio-economic status (U = 172.50, *P* = 0.091), and working memory (U = 204.00, *P* = 0.326) (Table 2).

### Supplemental analysis

To check if the differences in ToM abilities persist even when vocabulary is matched between groups, we repeated the analyses selecting a control sample that did not differ from the clinical sample for vocabulary (*P* = 0.814). Even in this sample, we found that healthy control children had higher ToM skills measured via mental SS (*P* = 0.049) and SF (*P* = 0.003). We found that children with NT1 performed better than healthy controls, equally good at vocabulary, in physical SS (*P* = 0.027) (Supplementary Table 1).

## Discussion

This is the first study aimed to investigate the existence of ToM impairment in children with NT1 and to examine the correlations between clinical variables and socio-cognitive skills. Our findings revealed that children and adolescents with NT1 have lower performances in ToM, assessed via mental SS and SF, but not in other cognitive skills, assessed via physical SS and working memory compared to healthy controls.

Assessing the other control variables, children with NT1 had lower vocabulary skills compared to healthy controls. To rule out the possible effect of this finding on ToM, we decided to control by matching patients and healthy subjects for vocabulary skills. Hence, the ToM performance of NT1 patients was still worse than healthy controls. However, NT1 patients had a higher score at the physical SS. Therefore, it is possible that NT1 patients showed poor results in vocabulary since it is a time-constrained task, in line with NT1 patients’ longer reaction times and slowness in cognitive tasks compared with healthy controls.^42,43^ These results highlight the existence of a specific ToM impairment in children with NT1 that is not driven by more general cognitive difficulties. Moreover, patients showed a higher perception of loneliness compared to healthy children. This finding fits with developmental literature showing impairment in the social functioning of narcoleptic children that could derive from a coping strategy to inhibit or suppress social communication that usually triggers cataplexy.^14,44^ Together, our findings support the hypothesis that NT1 significantly affects overall functioning, interfering with social relationships and competencies, resulting in a higher feeling of loneliness and in the impairment of ToM skills that are known to develop during childhood within the context of social relationships.^23,24^

ToM deficits in NT1 could be explained by the early onset of NT1 that may interfere with normal psychomotor development, altering some cognitive domains, including ToM, with milder impairment than other disorders, such as autism spectrum and schizophrenia, that are widely known to exhibit ToM deficits.^45,46^ In contrast to this hypothesis, we found no correlation between ToM impairment and the age of onset of narcolepsy or the duration of illness. Nevertheless, it is necessary to consider that the median diagnosis latency in our patients was 0.65 years. These data make our sample a particular pool of patients given that the literature showed that in Europe, the average delay in diagnosis of NT1 is 10 years.^47^ The earlier diagnosis and treatment might have reduced the neuropsychological impact of NT1 in our sample.

We found a negative correlation between verbal and visual ToM and symptoms’ severity that suggests that poorer ToM ability would result in greater exposure to the consequences of emotional stimuli, including cataplexy. Accordingly, NT1 patients are known to have a reduced expression of their own emotions, possibly as a coping strategy to inhibit or suppress those behaviours that usually trigger cataplexy.^14^ Recent studies on the neural correlates of cataplexy in children with NT1 showed that laughter episodes with cataplexy are accompanied by increased neural activity in a bilateral network that includes cortical and subcortical regions involved in processing saliency and emotions, encompassing the anterior insular cortex, the amygdala, the VMPFC and the ventral striatum.^48^ Concerning laughter without cataplexy, patients with NT1 showed stronger metabolic demand of a brain network encompassing the motor-premotor cortex and subcortical structures, namely the left subthalamic nucleus/zona incerta and ventrolateral and ventroposterior thalamic nuclei, when compared to healthy subjects. In addition, reduced activation of cortical-subcortical regions involved in humour detection and appreciation (the mesolimbic reward areas, the ventromedial prefrontal cortex, the temporo-occipital junction, the amygdala and the periaqueductal grey) has been found.^49^ These findings suggest a possible compensatory mechanism to avoid cataplexy by voluntarily inhibiting the affective component of humour.

Focusing on the clinical aspects of NT1, evidence demonstrated dysregulation of REM sleep,^50^ and it is worth noting how dream experiences during REM sleep can help emotional regulation and provide a tool to simulate a new scenario with elements of emotional mastery.^51^

Moreover, poor sleep causes impairments in executive functions (EF) and emotional information processing, both of which are associated with poor ToM ability.^52^

Finally, it is also possible to explain the negative correlation between symptoms’ severity and ToM in the direction that milder symptoms allow better participation in social situations prompting the development of ToM. Our data seem to confirm this evidence; specifically, we found that lower symptom severity is associated with higher perceived social inclusion. This result fits with the literature showing that relevant social impairment affected 45% of paediatric patients with NT1.^44^ Interestingly, our correlation analyses reveal that lower diagnosis latency is associated with higher perceived social inclusion and remarks the importance of an early NT1 diagnosis to reduce the disease burden and associated socio-cognitive impairment.^53^

Finally, the correlations of symptoms’ severity with both social inclusion and ToM could also be explained by the fact that ToM skills are known to have a key role in children’s adjustment when facing new social contexts.^54^ Thus, lower ToM may have in turn worsened NT1 children’s social functioning impairment and the inhibition of their emotion-expressive behaviour.

NT1 is a sleep disorder with a complex interplay of neurological, endocrinological and neuropsychiatric symptoms that can negatively impact patients’ overall outcome, especially during childhood.^1^

The results of our study show that children with NT1 have reduced ToM abilities compared with healthy subjects and, thus, greater difficulties in relating with and inferring other’s behaviour. It is still unclear whether NT1 plays a role in the pathogenesis of ToM impairment or is just a coexisting condition. A recent study showed that paediatric-onset craniopharyngioma patients with hypothalamic lesions were less able to identify the correct emotional content of vocal expressions and to infer others’ thoughts, feelings and intentions compared to healthy controls.^55^ However, there is still no evidence for a possible role of the hypocretinergic system in developing these cognitive functions.

Our results have important theoretical implications that fit the literature exploring emotional processing in NT1. Indeed, these findings provide evidence that behavioural manifestation of the NT1 neural alterations involving emotional processing could be detected through tasks assessing a socio-cognitive ability, namely ToM, that is more sensitive to individual differences than emotion recognition.^56^

We hypothesize that childhood-onset NT1 may interfere with the normal development of ToM and that subsequently, ToM abilities may modulate the severity of NT1 symptoms by providing a greater ability to avoid cataplexy.

The present study is not exempt from limitations. Indeed, a larger sample is needed to confirm our results. In addition, we adopted a cross-sectional design, further research should investigate how the trajectory of NT1 symptoms and ToM skills interact over time, adopting a longitudinal design.

In addition, our study significantly contributes to the understanding of emotional processing impairment in NT1 children. This opens the way for future studies to expand the scope of investigation by considering additional socio-cognitive measures, such as emotion recognition, and to investigate whether children NT1 have a specific impairment of advanced ToM or a broader impairment of emotional processing including emotion recognition.

Besides the theoretical implications, it leads the way for future studies to develop and test the efficacy of ToM training programmes aimed at improving ToM skills in NT1 patients and subsequently enhancing their social competencies.^29,57^ Moving the research in this direction would have important implications for improving the quality of life of NT1 patients, given that their impairments in social functioning could impact, in a broader sense, their psychological well-being.

## Acknowledgements

This work was developed within the framework of the DINOGMI Department of Excellence of MIUR 2018–2022 (law 232/2016). This work was supported by the Italian Ministry of Health, 5 × 1000 project 2017 “SOLAR: Sleep disorders in children: an innovative clinical research perspective.

## Supplementary Material

fcae063_Supplementary_Data

## Data Availability

The data that support these findings are available from the author upon reasonable request.
