# Supplementary material for: Theory of Mind impairment in childhood narcolepsy type 1: a case–control study
Source: Brain Commun. 2024 Feb 27;6(2):fcae063. doi: 10.1093/braincomms/fcae063 (PMC10935651; doi:10.1093/braincomms/fcae063)
Supplement: fcae063_Supplementary_Data [file fcae063_supplementary_data.pdf]

Supplementary Table 1 Differences within Theory of Mind and control variables between NT1 and healthy controls matched for age, sex, and vocabulary

	Healthy controls			NT1			
	Median	Q1 – Q3	Range	Median	Q1–Q3	Range	p-value
Age	11.3	9.5 – 12.4	8.1 – 13	11.4	9.11 – 12.1	8 – 13.5	.981
Vocabulary	16.5	8.74 – 22.25	6 – 34	16.00	12 – 21	5 – 31	.814
WM	2.5	2 – 3	1 – 4	3.00	3 – 3	1 – 5	.067
physical SS	3	2.75 – 4	0 – 6	4.00	3 – 5	0 – 6	.027*
mental SS	7	6 – 8.25	5 – 9	5.00	4 – 8	2 – 9	.049*
SF	8	6.75 – 9	4 – 12	6.00	3.75 – 7	2 – 10	.003**

*** p < .001. ** p < .01. * p < .05 SES = Socio-Economic Status. Voc = Vocabulary. WM = Working Memory. SS = Mental Strange Stories. SF = Silent Film. LSDQ = Loneliness & Social Dissatisfaction Questionnaire. SI = Social Inclusion. DMF = Difficulty of Making Friends. PL = perceived Loneliness. SASC = Social Anxiety Scale for Children. FNE = Fear for Negative Evaluation. SAD = Social Avoidance and Distress.

Supplementary Fig. 1 Strange Stories task

A

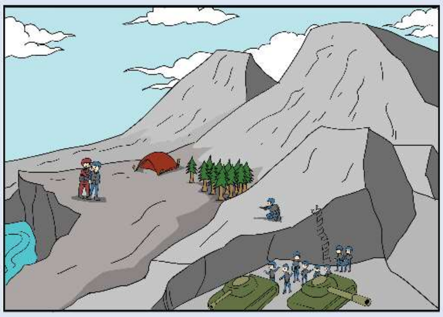

During the war, the red army arrests a member of the blue army and wants to know where their tanks are. The red army knows they are at the sea or mountain. The red army members also know that the prisoner does not want to tell where the tanks are and will surely lie. The prisoner is courageous and diligent and will ensure they do not find the tanks. The Blue Army tanks are located on the mountain. When the members of the Red Army ask him where they are, he replies, "On the mountain."

Question: **Why does the prisoner say so?**

Correct answer example: **He knows that the enemy army will not believe him**

B

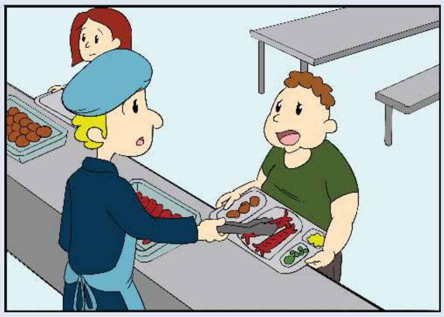

Francesco is always hungry. Today at school, there is his favorite meal: sausages and beans. Since he is a very greedy boy, he would like to have more sausages than anyone else, even though he knows that when he gets home, his mom will have prepared a delicious meal for him! But everyone is allowed to receive two sausages and no more. So when Francis is served, he says, " Please, can I have four sausages? When I get home, they won't feed me!"

Question: **Why does Francesco say that?**

Correct answer example: **He wants to pity the cook to get more sausages**

C

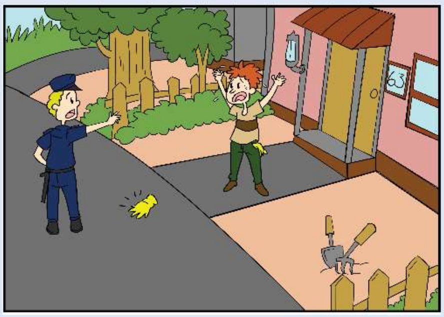

A thief has just robbed a store and is running away. As he is running home, a policeman sees him losing a glove. The policeman does not know he is a thief and wants to tell him that he dropped the glove. However, when the policeman shouts to the thief, "Hey you, stop!" the thief raises his arms and admits that he committed the shoplifting.

Question: **Why does the thief say that?**

Correct answer example: **Because he believes that the policeman realized that he had committed the theft**

D

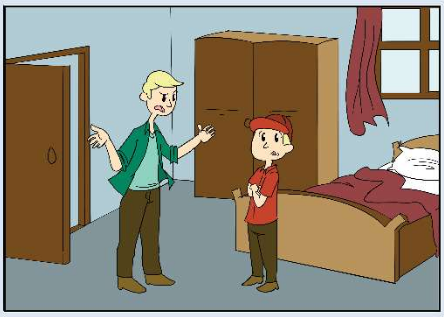

Simone is a great liar. His brother, Giacomo, knows very well that Simone never tells the truth! Just yesterday, Simone stole his ping-pong paddle. Giacomo knows that Simone hid it somewhere, but he can't find it. Giacomo is furious. So, he says to Simone, "Where is my ping-pong paddle? You must have put it in the closet or under your bed because I've looked everywhere and didn't find it. Where is it? In the closet or under your bed?" Simone replies that the paddle is under his bed.

Question: **Why will Giacomo look for the paddle in the closet?**

Correct answer example: **Because he knows that his brother is a liar**

E

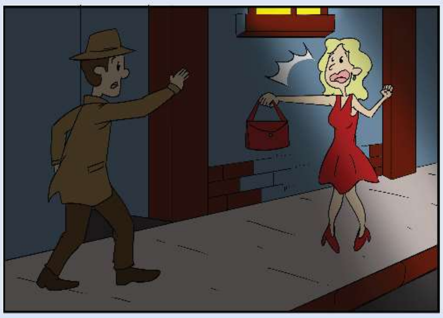

Late at night, old Mrs. Bianchi is walking home. Mrs. Bianchi does not like walking home alone at night because she fears someone might attack and rob her. She is indeed a very nervous person. Suddenly a man emerges in the dark. He wants to ask Mrs. Bianchi what time it is, so he approaches her. When Mrs. Bianchi sees the man walking toward her, she trembles and says, "Take my bag, but please don't hurt me!"

Question: **Why does Mrs. Bianchi say that?**

Correct answer example: **She does not know that the man does not intend to rob her**

(A) and (D) Double bluff stories. (B) Persuasion story. (C) and (E) Misunderstanding stories.
